# Endoscopic Mucosal Incision for Removal of a Deeply Embedded Esophageal Fish Bone: A Case Report

**DOI:** 10.1002/ccr3.73167

**Published:** 2026-07-15

**Authors:** Liang Deng, Liang Luo, Su‐Bi Feng, Meng‐Ru Wu, Xiao‐Ning Lian

**Affiliations:** ^1^ Department of Gastroenterology Lunjiao Hospital Foshan City Guangdong PR China

**Keywords:** computed tomography, endoscopic management, endoscopic mucosal incision, fish bone, foreign body

## Abstract

Deeply embedded esophageal foreign bodies may not be visible on conventional endoscopy. Careful CT evaluation can guide successful endoscopic mucosal incision when advanced endoscopic imaging modalities are unavailable, potentially avoiding surgery in selected patients.

## 1 Introduction

Esophageal foreign body ingestion is a common clinical problem encountered in emergency and gastroenterology practice. Although many ingested objects pass spontaneously, sharp foreign bodies such as fish bones are associated with a higher risk of impaction and serious complications, including perforation, mediastinitis, and vascular injury if not promptly managed [1, 2]. Early diagnosis and timely removal are therefore essential to reduce morbidity. Endoscopy is the first‐line diagnostic and therapeutic modality and achieves high success rates in most cases [2]. However, in a subset of patients, particularly those with delayed presentation, foreign bodies may become embedded within the esophageal wall or migrate beyond the lumen, making detection by conventional endoscopy challenging [3]. Imaging plays a crucial role in these situations, with computed tomography (CT) having been shown to provide superior diagnostic accuracy and precise localization [4, 5]. In addition, CT can evaluate the relationship between the foreign body and adjacent structures, which is essential for planning safe intervention.

We report a case of a fish bone deeply embedded in the esophagus that was not visible on endoscopy and was successfully removed using CT‐guided endoscopic mucosal incision.

## 2 Case History/Examination

A 59 year old female patient experienced a sore throat after eating fish. Four days later, her condition did not improve and she visited the ENT outpatient department in our district hospital. On examination, the patient reported no fever, dysphagia, or chest pain. Physical examination was unremarkable, and vital signs were stable. Given the persistent sore throat and history of fish consumption, an esophageal foreign body was suspected and she was taken to the gastroenterology department for further examination.

## 3 Differential Diagnosis, Investigations and Treatment

**FIGURE 1:**
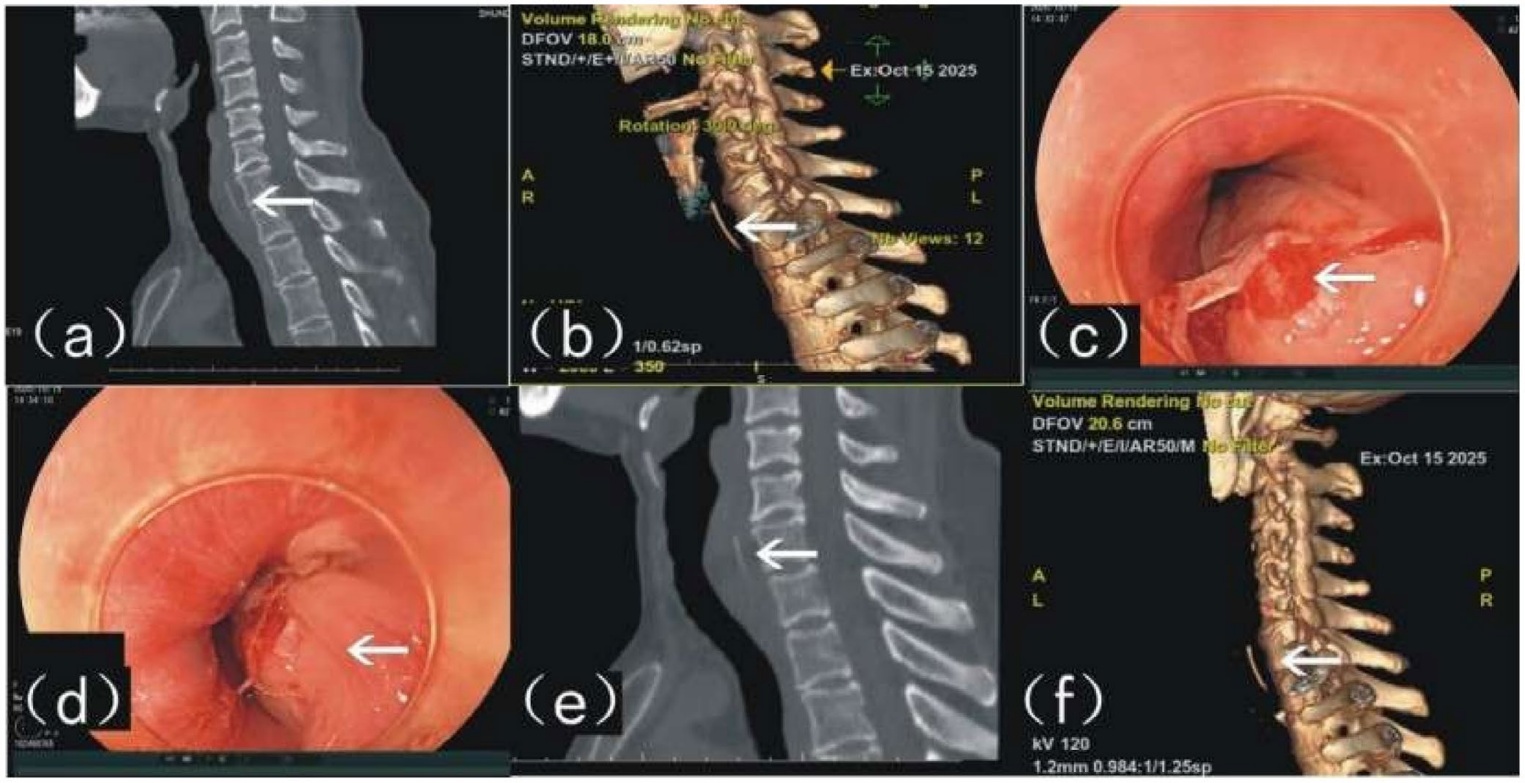
(a) The CT scan revealed a fish bone like foreign body in the upper esophagus (white arrow). (b) Three‐dimensional CT reconstruction confirmed the foreign body anterior to the sixth and seventh cervical vertebrae (white arrow). (c) The Upper GI endoscopy revealed swelling at the entrance of the esophagus, with the friable mucosa and white secretions. (d) Submucosal tumor‐like swelling of the esophagus. (e) Repeat CT confirming persistence of the foreign body (white arrow). (f) The repeat CT three‐dimensional reconstruction showing unchanged location and size of the foreign body.

**FIGURE 2:**
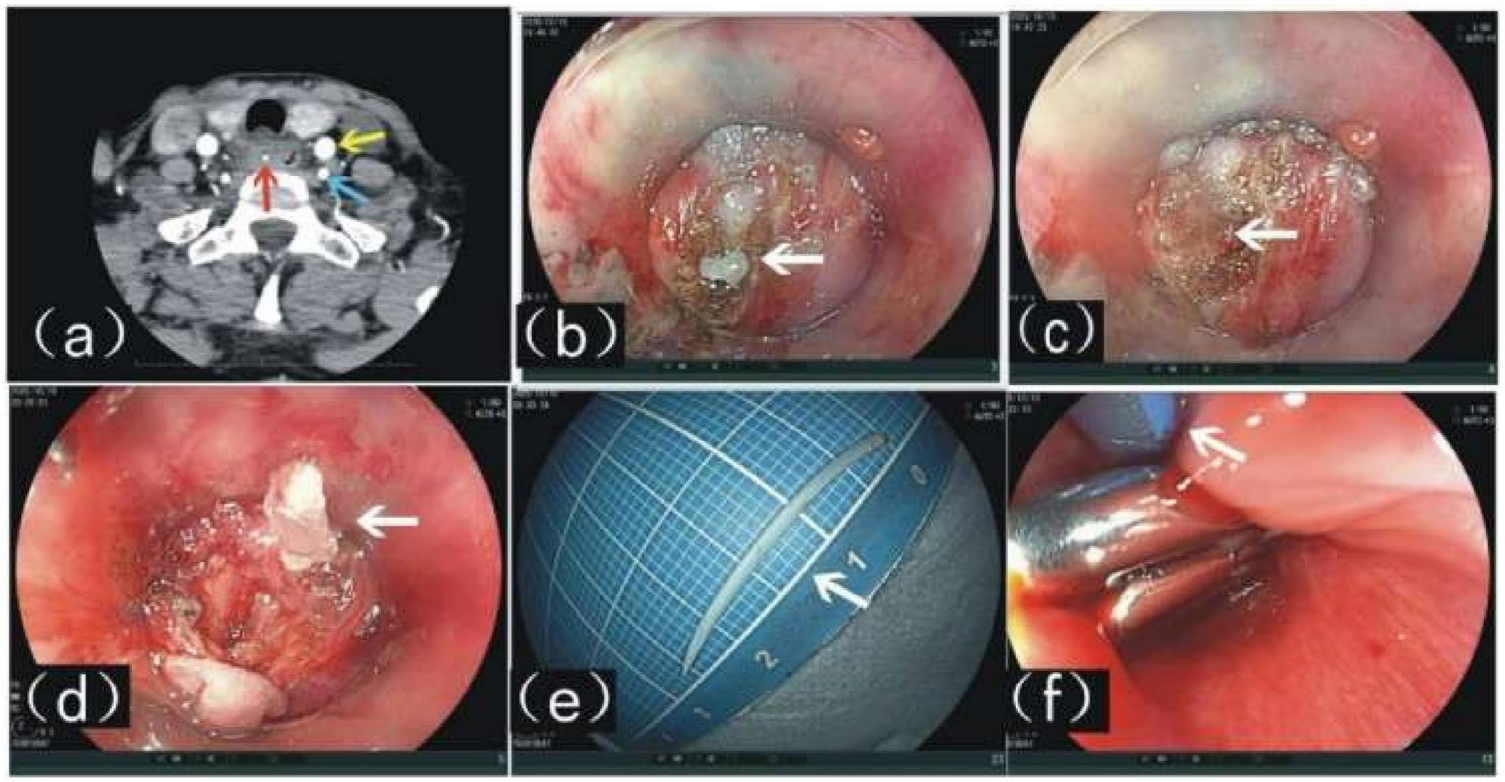
(a) Contrast‐enhanced CT demonstrating the foreign body (red arrow) and its relationship to adjacent vessels, including the common carotid artery (yellow arrow) and vertebral artery (blue arrow). (b) Endoscopic mucosal incision with drainage of purulent material (white arrow). (c) Identification of the embedded fish bone within the abscess cavity (white arrow). (d) Enlargement of the incision to expose the foreign body (white arrow). (e) Extracted fish bone measuring approximately 2.5 cm (white arrow). (f) Post‐procedural closure with clips and placement of a gastric tube (white arrow).

A CT scan demonstrated linear hyper‐dense structure consistent with a fish bone at the level of the sixth and seventh cervical vertebrae in the esophagus (Figure 1a,b). The patient was then admitted for treatment. Upon admission, a blood test showed a slightly elevated white blood cell count of 9.87 × 10^9^/L (normal range: 3.5–9.5 × 10^9^/L). An upper GI endoscopy was performed and although the foreign body was not seen, the cervical esophageal mucosa appeared swollen with a small amount of pus oozing out (Figure 1c,d). As endoscopic ultrasound expertise was not available at our hospital, a repeat CT scan was performed to confirm persistence and reassess the location of the foreign body before further intervention planning (Figure 1c,d). Subsequently, contrast enhanced CT was performed to evaluate the relationship between the foreign body and adjacent vascular structures (Figure 2a). The contrast‐enhanced CT confirmed the absence of vascular involvement and showed the retained foreign body with an associated local abscess (Figure 2b,c). An endoscopic mucosal incision was performed under general anesthesia with tracheal intubation in the operating room to remove the embedded foreign body. The mucosa and submucosa were incised layer by layer, along the abscess. The muscular layer was then gradually explored, incised, and separated (Figure 2d). Pus was visible and served as a useful guide to the lesion. After gradually incising and expanding the abscess, a grayish‐white suspected end of the foreign body was exposed measuring 2.5 cm in length (Figure 2e). It was then clamped using forceps and slowly pulled out.

## 4 Outcome and Follow Up

The abscess cavity was continuously irrigated with metronidazole and normal saline. The wound was then clamped with clips, and a gastric tube was inserted to provide enteral nutrition (Figure 2f). The procedure lasted 70 min.

**FIGURE 3:**
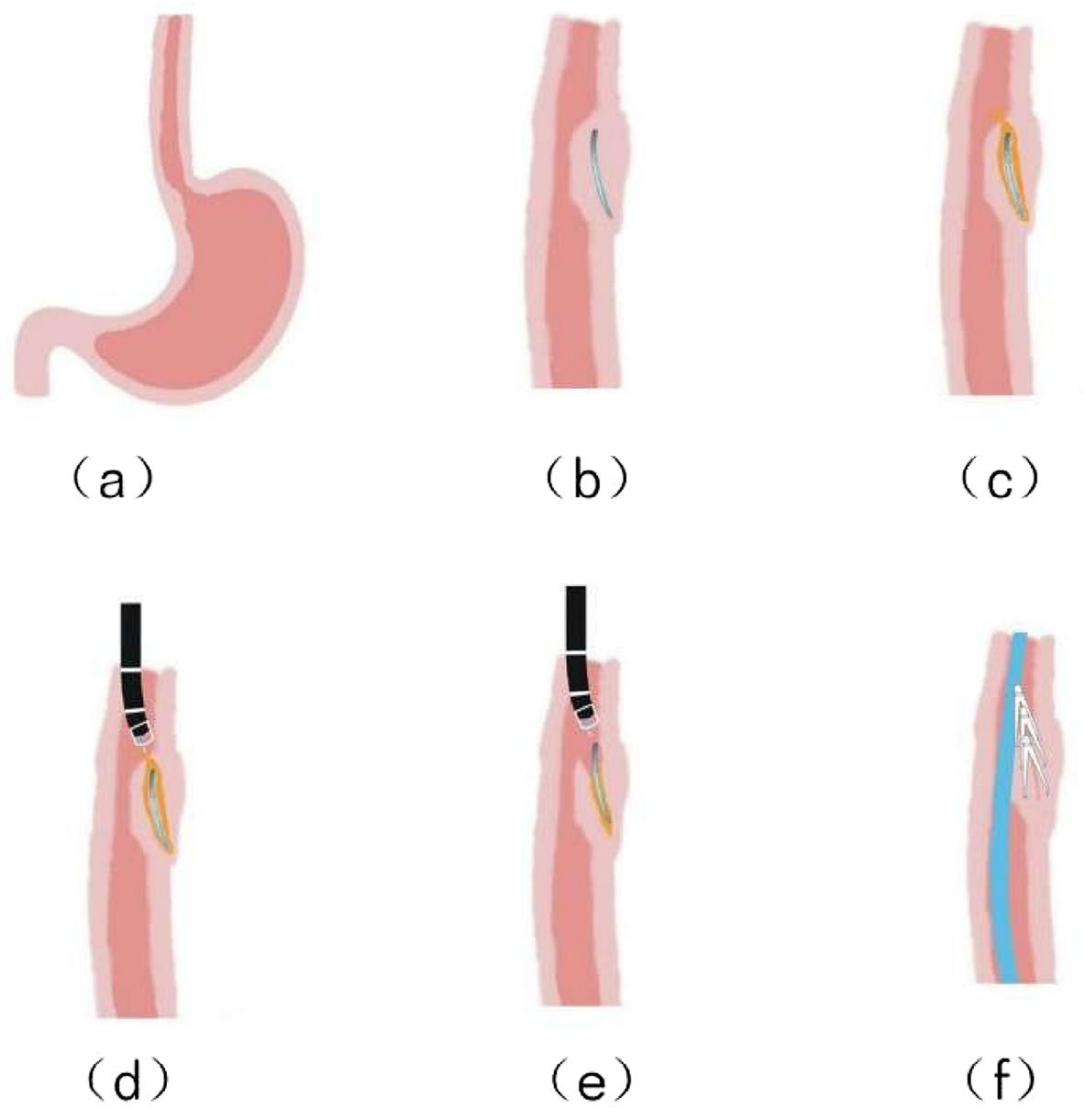
Schematic diagram of the process of removing the foreign body from the mucosa under endoscopy. (a) Normal esophagus. (b) The foreign body penetrates the mucosa and becomes embedded in the submucosa, rendering it invisible on endoscopy. (c) Local infection leads to esophageal swelling and abscess formation. (d) Stepwise endoscopic mucosal and submucosal incision at the site of purulent discharge to expose the underlying layers. (e) Identification and removal of the foreign body after adequate exposure. (f) Closure of the mucosal defect and placement of a gastric tube.

Following an uneventful 1 week inpatient recovery, the gastric tube was removed after the patient demonstrated safe resumption of oral intake. During a telephone follow up 1 week after discharge, the patient reported no significant discomfort. At 1 month after discharge the patient remained asymptomatic, with no evidence of recurrent symptoms (Figure 3).

## 5 Discussion

Deeply embedded esophageal foreign bodies present a diagnostic and therapeutic challenge, particularly when not visible on conventional endoscopy. Delayed presentation, as in this case, increases the risk of transmural migration and complications such as perforation or mediastinitis [2, 6]. Fish bones are commonly ingested sharp foreign bodies and have a high propensity to penetrate the esophageal wall [1, 7]. Once embedded, they may become covered by mucosa, limiting detection by standard endoscopy. CT is therefore essential, as it provides accurate localization and helps assess surrounding structures [8]. Contrast‐enhanced CT is particularly useful for evaluating proximity to major vessels and guiding safe intervention.

Although endoscopic removal is first‐line, surgery has traditionally been required when foreign bodies are deeply embedded or not visualized [9]. However, advances in endoscopic techniques have enabled minimally invasive alternatives. Endoscopic mucosal incision has been reported as an effective method for removing submucosal foreign bodies [10], while techniques such as endoscopic ultrasound guidance and gel immersion further improve localization and safety [11, 12, 13].

Compared with surgery, this approach may offer the advantages of reduced invasiveness and faster recovery in carefully selected patients but requires advanced endoscopic expertise. This case suggests that CT‐guided endoscopic mucosal incision can be a feasible minimally invasive alternative to surgery for selected patients, particularly when careful imaging confirms the absence of adjacent vascular involvement. Early imaging assessment, appropriate patient selection, and advanced endoscopic techniques are essential for successful management and favorable outcomes.

## Author Contributions

**Meng‐Ru Wu:** methodology, data curation, formal analysis, writing – review and editing. **Liang Luo:** conceptualization, investigation, methodology, data curation, validation, writing – review and editing, writing – original draft. **Xiao‐Ning Lian:** writing – review and editing, investigation, data curation, formal analysis. **Liang Deng:** conceptualization, project administration, supervision, investigation, methodology, writing – review and editing, writing – original draft. **Su‐Bi Feng:** visualization, data curation, formal analysis, writing – review and editing.

## Funding

The authors have nothing to report.

## Ethics Statement

The authors have nothing to report.

## Consent

Written informed consent for publication of this case and accompanying images was obtained from the patient.

## Conflicts of Interest

The authors declare no conflicts of interest.

## Data Availability

The data that support the findings of this study are available from the corresponding author upon reasonable request.
